# MiR-150 promotes cellular metastasis in non-small cell lung cancer by targeting FOXO4

**DOI:** 10.1038/srep39001

**Published:** 2016-12-15

**Authors:** Hui Li, Ruoyun Ouyang, Zi Wang, Weihua Zhou, Huiyong Chen, Yawen Jiang, Yibin Zhang, Hui Li, Mengting Liao, Weiwei Wang, Mao Ye, Zhigang Ding, Xueping Feng, Jing Liu, Bin Zhang

**Affiliations:** 1 The State Key Laboratory of Medical Genetics & School of Life Sciences, Central South University, Changsha, 410078, China; 2 Department of Respiratory Medicine, Respiratory Disease Research Institute, Second XiangYa Hospital of Central South University, Changsha, 410011, China; 3 Department of Histology and Embryology, Xiangya School of Medicine, Central South University, Changsha, 410013, China; 4 Molecular Science and Biomedicine Laboratory, State Key Laboratory for Chemo/Biosensing and Chemometrics, College of Biology, College of Chemistry and Chemical Engineering, Collaborative Innovation Center for Chemistry and Molecular Medicine, Hunan University, Changsha, 410082, China; 5 Center for Experimental Medicine, the Third Xiangya Hospital, Central South University, Changsha, 400083, China; 6 Institute of Medical Sciences, Xiangya Hospital, Central South University, Changsha, 410078, China

## Abstract

Previous studies have shown that dysregulation of microRNA-150 (miR-150) is associated with aberrant proliferation of human non-small cell lung cancer (NSCLC) cells. However, whether miR-150 has a critical role in NSCLC cell metastasis is unknown. Here, we reveal that the critical pro-metastatic role of miR-150 in the regulation of epithelial-mesenchymal-transition (EMT) through down-regulation of FOXO4 in NSCLC. *In vitro*, miR-150 targets 3′UTR region of FOXO4 mRNA, thereby negatively regulating its expression. Clinically, the expression of miR-150 was frequently up-regulated in metastatic NSCLC cell lines and clinical specimens. Contrarily, FOXO4 was frequently down-regulated in NSCLC cell lines and clinical specimens. Functional studies show that ectopic expression of miR-150 enhanced tumor cell metastasis *in vitro* and in a mouse xenograft model, and triggered EMT-like changes in NSCLC cells (including E-cadherin repression, N-cadherin and Vimentin induction, and mesenchymal morphology). Correspondingly, FOXO4 knockdown exhibited pro-metastatic and molecular effects resembling the effect of miR-150 over-expression. Moreover, NF-κB/snail/YY1/RKIP circuitry regulated by FOXO4 were likely involved in miR-150-induced EMT event. Simultaneous knockdown of miR-150 and FOXO4 abolished the phenotypic and molecular effects caused by individual knockdown of miR-150. Therefore, our study provides previously unidentified pro-metastatic roles and mechanisms of miR-150 in NSCLC.

Lung cancer is the leading cause of cancer-associated deaths worldwide, with an estimated 224,210 new cases and 159,260 deaths in 2014 in the United States^1^. NSCLC is the most common lung cancer, accounting for approximately 87% of all cases, and it is insensitive to chemotherapy and/or radiation therapy compared with small cell lung cancer (SCLC). Metastasis is the most deadly aspect of NSCLC. NSCLC patients often relapse and develop metastases after surgery, radiation therapy, and/or chemotherapy, resulting in an overall five-year survival rate of less than 18%^2^. Therefore, to improve the diagnosis, prognosis and drug-targeted therapies for NSCLC, it is important to screen metastases-related genes and explore their underlying molecular mechanisms^3^.

MicroRNAs are a class of noncoding RNAs, 18–25 nucleotides (nt) in length, encoded by distinct genes and undergoing a sophisticated process to mature to evolutionarily conserved single-stranded forms. Generally, miRNAs bind to the 3′ untranslated region (3′UTR) of their target mRNAs and suppress protein production by translational repression or induction of mRNA degradation. Aberrant miRNA expression profiles play a critical role in tumorigenesis and tumor development, and may serve as biomarkers for tumor diagnoses and therapies^4^,^5^. MiR-150 was initially identified as a hematopoietic cell-specific miRNA, affecting the differentiation of numerous hematopoietic cell lineages^6^. Recent studies demonstrated that miR-150 was also involved in human tumors, including hematopoietic malignancies such as lymphoma and leukemia^6^ and solid tumors. However, miR-150 may function as either an oncogene or a tumor suppressor in different tumor types, which is dependent on the expression levels of it and the action of its target genes in certain tumor types. To date, the functions of miR-150 in relation to proliferation phenotypes of tumor cells have been extensively studied and a sequence of targets have been identified. For example, as an oncomiR, miR-150 is significantly over-expressed and promote cell proliferation in lung cancer (identified targets: p53 and BAK1)^7^,^8^, breast cancer (target: P2X7)^9^ and gastric cancer (target: EGR2)^10^. On the contrary, miR-150 was found to be down-regulated in malignant pancreatic tissues. Over-expression of miR-150 decreased tumor cell growth and clonogenicity via targeting MUC4 *in vitro*^11^. Although miR-150 plays a metastasis suppressor role in esophageal squamous cell carcinoma and hepatocellular carcinoma via targeting ZEB1 and GAB1, respectively^12^,^13^, the exact role (anti- or pro- metastasis) of miR-150 in lung cancer metastasis *in vitro* and *in vivo* and potential metastasis-associated molecular mechanisms remain poorly understood.

The FOXO (forkhead box O) family is composed of four members: FOXO1, FOXO3, FOXO4 and FOXO6 that are characterized by a conserved winged-helix DNA-binding domain called the ‘forkhead box’. FOXO proteins use the forkhead box domain to bind as monomers to the consensus sequence (5′-TTGTTTAC-3′) and hence negatively or positively regulate gene expression, depending on the promoter context and extracellular conditions^14^,^15^,^16^. FOXO proteins are subject to multiple posttranslational regulations typically from the PI3K-AKT/SGK pathway (phosphorylation), the stress-activated JNK pathway (phosphorylation) and other posttranslational modifications such as acetylation and methylation, which decide on the subcellular localization (nuclear-cytoplasmic shuttling), DNA binding affinity and transcriptional activity^17^. However, emerging evidence shows that the mRNA expression levels of FOXOs vary between normal and tumor tissues and that an inverse correlation between the expression of FOXOs and miRNAs was observed in a panel of tumors and tumor cell lines, which implies that that expression levels of FOXO transcripts are tightly regulated by the miRNA networks^16^,^18^. A growing number of miRNAs have been shown to target FOXO4. For example, aberrant up-regulation of miR-499-5p and miR-1274a promote tumor metastasis in colorectal and gastric cancer respectively by targeting FOXO4^19^,^20^. Recently, miR-150 was found to promote tumor cell proliferation by targeting FOXO4 in cervical carcinoma^21^. However, whether miR-150 targets FOXO4 to promote NSCLC metastasis is unknown and will be investigated in this study.

In this study, we first demonstrated miR-150 directly targets the 3′UTR of FOXO4 to inhibit its expression using a dual-luciferase reporter assay. We further investigated the expression of miR-150 and FOXO4 in NSCLC cell lines and tissue samples, and analyzed the association of their expression with metastatic characteristic of NSCLC patients. Furthermore, we validated that miR-150 promoted tumor cells metastasis and EMT via FOXO4-mediated regulation of NF-κB/snail/YY1/RKIP circuitry, which consequently triggered the down-regulation of E-cadherin. Our results suggest that miR-150 is a novel metastasis marker in NSCLC and miR-150-FOXO4 signaling might be a potential target for therapy in NSCLC metastasis.

## Materials and Methods

### Cell culture, plasmids and transfections

Human NSCLC cell lines (H460, A549, H1299, 95 C, 95D) and human normal lung cell line (MRC-5) were purchased from the Cell Bank of Type Culture Collection of the Chinese Academy of Sciences, Shanghai Institute of Cell Biology. MRC-5 is a human lung fibroblast cell line, 95C/D are human giant cell lung carcinoma cell lines, H1299 and H460 are human large-cell lung carcinoma cell lines, A549 is a human lung adenocarcinoma cell line. A549, 95 C, 95D and MRC-5 cell lines were grown in DMEM medium (Gibco, USA). H460 and H1299 cell lines were grown in RPMI-1640 medium (Gibco, USA) supplemented with 10% fetal bovine serum (Gibco, USA) and penicillin 100 (U/ml)/streptomycin (100 μg/ml) at 37 °C in a humidified atmosphere with 5% CO_2._

Pre-miR-150 plasmids and paired null vectors were constructed by our lab^22^. The MiR-150 inhibitor, inhibitor negative control (NC-inhibitor), small interfering RNA (siRNA) targeting FOXO4, and an unrelated sequence was used as a negative control of siRNA (siNC) were synthesized by GenePharmaCo., Ltd (Shanghai, China). miR-150, miR-421, miR-664a-3p, miR-499a-5p mimics and miRNA mimic NC were synthesized by Ribobio Technology Co., Ltd. (Guangzhou, China). The sequence of materials is listed in Supplementary Table 1.

For transfection, experimental protocols were performed according to our previously published protocols^22^. MiR-150 inhibitor/NC inhibitor or siFOXO4/NC siRNA were transfected into cells using Lipofectamine 2000 (Invitrogen, Carlsbad, USA) according to the manufacturer’s instructions. MiRNA mimics and miRNA mimic NC were transfected into cells using ribo FECT ™ CP Transfection Kit (Ribobio Technology Co., Ltd, Guangzhou, China) according to the manufacturer’s instructions. After a 48 h transfection, cells were used for further experiments. Pre-miR-150 plasmid/control vector were transfected into NSCLC cells and screened for 3–4 weeks with 1 μg/ml puromycin after a 48 h transfection.

### Human NSCLC tissue samples

16 cases of human non-neoplastic lung tissues and 36 cases of human NSCLC lung tissues including 19 cases of non-metastatic NSCLC tissues (adenocarcinoma in 4 cases and squamous carcinoma in 15 cases) and 17 cases of metastatic NSCLC tissues (adenocarcinoma in 3 cases, squamous carcinoma in 13 cases and adeno-squamous carcinoma in 1 cases) were collected from the Second Xiangya Hospital of Central South University (Changsha, Hunan, China). This study was carried out after approval by the Ethics Committee of the Second Xiangya Hospital and obtaining informed consent from all subjects. The methods in treating tissues were carried out strictly in accordance with institutional policies and approved guidelines of experiment operations.

### RNA extraction and quantitative reverse transcription-PCR (qRT-PCR)

For miRNA or mRNA content detection, total RNA was extracted from cells or tissues using Trizol (Invitrogen, USA) according to the manufacturer’s instructions. cDNA was synthesized via RevertAid First Strand cDNA Synthesis Kit (Thermo Scientific, USA). The reverse transcription reaction for miR-150 was carried out with stem-loop RT primers (RiboBio, China). QRT–PCR was performed using a SYBR Green Master Mix (Bio-Rad, USA). Relative expression was determined using U6 snRNA (primers from RiboBio, China) as an internal control for miR-150 and GAPDH as an internal control for FOXO4. The primers used are listed in Supplementary Table 1.

### Wound-healing assays

Experimental protocols were conducted according to our published protocol^23^. Transfected cells were seeded in 6-well cell culture plates and cultured until 90–100% confluent in complete medium. Cell monolayers were wounded using a P200 pipette tip held vertically, and the layer was washed several times with PBS to remove cell debris. Images were captured from the same region at 0, 24 and 48 h for each wound.

### Transwell migration assays

Experimental protocols were conducted according to our published protocol^23^. Cells (4 × 10^5^ cells/well) were transfected and seeded into the top chamber of 24-well Transwell plates (Corning Incorporated, USA) containing 1% FBS medium. Complete medium was added to the bottom chamber. After a 24 h incubation in normal conditions, the cells on the upper side of the filters were swabbed with a cotton swab, and the cells on the lower surface of the filters were fixed with 4% paraformaldehyde for 20 min and stained with 1% crystal violet for 30 min. Images were captured by light microscope, and average migrating cells from five independent fields were counted.

### Western blot analysis

Experimental protocols were conducted according to our published protocol^23^. Cells or tissues were lysed with RIPA buffer (150 mM NaCl, 25 mM Tris-HCl, pH 7.4, 0.1% SDS, 1% Triton X-100, 1% deoxycholate, 2 mM EDTA) that contained a protease or phosphatase inhibitor mixture (Roche, France); 50 μg of proteins were separated by SDS-PAGE followed by immunoblotting with specific antibodies (Supplementary Table 2).

### F-actin staining

The procedure used has been described in detail in our earlier study^24^. Briefly, A549 cells were seeded on coverslips in 6-well dishes and transfected with the miR-150 plasmid and the corresponding control vector for 48 h and then were fixed, permeabilized, blocked and stained, according to the manufacturers’ instructions, with Alexa Fluor 488 Phalloidin (Invitrogen, Carlsbad, USA). Images were captured by the Leica SP5 II scanning confocal microscope (Leica, Bannockburn, USA).

### Dual luciferase reporter assay

The procedure used has been described in detail in our earlier study^22^. The human FOXO4 wild type or mutated 3′-UTR sequence containing the miR-150 binding site was cloned into the psiCHECK-2 vector. The primer sequences are listed in Supplementary Table 1. Cells were seeded into 24-well plates and co-transfected with miR-150 or control vector and wild-type or mutated *foxo4* 3′-UTR using Lipofectamine 2000. Both firefly and Renilla luciferase activities were measured after the 48 h transfection using the Dual-Luciferase Reporter 1000 Assay System (Promega, USA) and were detected by the GloMax TM 20/20 detection system (E5331, Promega, USA) according to the manufacturer’s instructions. Luciferase activities were normalized to Renilla luciferase.

### Soft agar colony formation assay

Experimental protocols were conducted according to our published protocol^24^. The bottom layer consisting of 0.5% low-melting-temperature agarose (BD) with Basal medium eagle medium (BME, Sigma) containing L-Glu and gentamycin was first formed on 6-well plates. Then, cells were diluted in BME with 10% FBS mixed with low-melting-temperature agarose, forming the top layer (5,000 cells per well). The cell colony formation numbers were counted (under light microscopy) after two weeks of cultivation.

### Tumorigenicity and tumor metastasis assays in nude mice

Nude mice, 4–6 weeks old, were purchased from the Shanghai Lab Animal Research Center (Shanghai, China) and cared for in the Experiment Animal Center of the Central South University (Changsha, China). For tumorigenicity assays, 2 × 10^6^ stably transfected cells in 0.2 ml RPMI-1640 medium were injected subcutaneously into the right upper back of the mice. The tumor volume for each mouse was measured every 3 days. Three weeks after the injections, tumor-bearing mice were sacrificed, and the size of the tumor was determined by caliper measurement. For tumor metastasis assays, 2 × 10^6^ cells in 0.2 ml were injected into the lateral tail vein of each mouse. Each group had five mice. After 30 days, the mice were sacrificed, and their lungs were examined for tumor metastases using Hematoxylin and Eosin (H&E) stains. All animal procedures were approved by the Animal Care and Use Committee of the third Xiangya Hospital of Central South University (Changsha, Hunan, China) and performed strictly in accordance with institutional policies and approved guidelines of experiment operations.

### Immunohistochemistry

The procedure used has been described in detail in our earlier study^23^. Briefly, the slides were deparaffinized in xylene and dehydrated. Slides were then heated in a microwave with citrate buffer (pH 6.0) for 15 minutes. After blocking the endogenous peroxidase activity with 3% H_2_O_2_ for 10 minutes, the slides were blocked with 10% normal goat serum for 30 minutes and then incubated with anti-FOXO4 antibody overnight at 4 °C. Staining was visualized with diaminobenzidine (DAB; Sigma-Aldrich). After staining, sections were counterstained with hematoxylin. The slides were then washed with PBS, dehydrated, cleared in xylene and mounted with permount.

### Statistical analyses

All statistical analyses were performed using SPSS 16.0 statistical software. 2-sided unpaired Student’s t-tests were used to determine the significance of the differences between the control and the experimental groups. A *p* < 0.05 was considered statistically significant.

## Result

### FOXO4 is a direct binding target of miR-150 in NSCLC

MiRNA exerts its effects mainly through the suppression of downstream target genes. Therefore, the targets of miRNA-150 were investigated. Using two computational miRNA target prediction tools (miRBase and TargetScan), several candidate targets with a relatively high prediction score, including FOXO4, c-myb, zeb1, 4.1 R and PI3KCB (Phosphoinositide-3-Kinase, Catalytic, Beta Polypeptide), were selected for further target identification. Based on the protein expression levels and previous reports, FOXO4 were picked up on account of the following reasons: (1) Among these molecules, only FOXO4 displayed dramatic reverse changed in its expression in response to altered expression of miR-150 (Supplementary Fig. S1); (2) Previously, the decreased expression of FOXO4 was associated with increased malignance in gastric and prostate cancers^25^,^26^, suggesting that FOXO4, as a putative tumor suppressor gene, is implicated in the development of human cancers. Furthermore, a comparison was made in their negative regulatory effects on FOXO4 protein expression between miR-150 and several other potential up-stream miRNAs of FOXO4 (miR-421, miR-664a-3p, miR-499a-5p). Exogenous expression of miR-150 showed positive potent inhibitory effect on the protein expression of FOXO4 with a significant statistical difference when compared to other miRNAs in NSCLC (Supplementary Fig. S2), implying that there is a critical miR-150-FOXO4 interaction in NSCLC.

**Figure 1:**
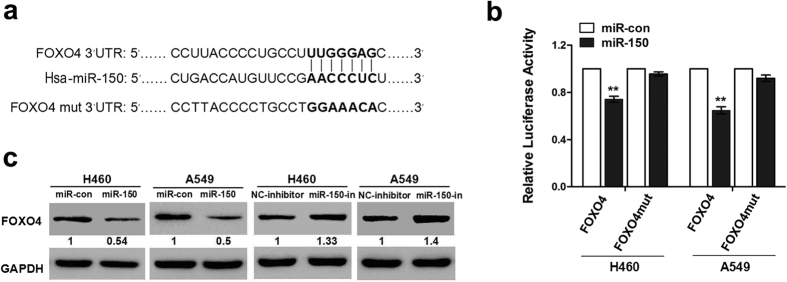
FOXO4 is a down-stream target of miR-150. (**a**) Sequences of the conserved Hsa-miR-150 binding sites in the 3′UTR of wild-type FOXO4 mRNA and a FOXO4 mutant containing seven mutated nucleotides in the 3′UTR of FOXO4 (FOXO4-mut-3′UTR). (**b**) Luciferase activity assays of the FOXO4-3′UTR or FOXO4-mut-3′UTR reporter in H460 and A375 cells transfected with the pre-miR-150 plasmid or control vector. Experiments were performed in triplicate, and data are expressed as the mean ± SD, **p < 0.01. (**c**) Western blot analysis showing the alteration of FOXO4 expression in H460 and A549 cells transfected with the pre-miR-150 plasmid, miR-150 inhibitor or their corresponding control vectors. GAPDH served as a loading control. A single band was detected at ~54 kDa, reflecting full-length expression. Fold change in FOXO4 protein level normalized to GAPDH is shown numerically and at the bottom of protein bands.

We further examined whether FOXO4 was a direct target of miR-150 in NSCLC cells using a dual-luciferase reporter assay. Transient co-transfection of pre-miR-150 plasmids and luciferase reporters carrying the FOXO4 3′UTR into H460 and A549 cells resulted in a markedly decreased activity of the luciferase reporters, compared to co-transfection of paired null vectors and luciferase reporters. However, this suppressive effect was abolished by the creation of a point mutation in the miR-150-binding region of in the FOXO4 3′UTR (Fig. 1a and b). Next, we confirmed the inverse relationship between the expression of FOXO4 and miR-150 in H460 cells and A549 cells. As shown in Fig. 1c, over-expression of miR-150 by pre-miR-150 plasmid inhibited FOXO4 expression, while inhibition of miR-150 by miR-150 inhibitor enhanced expression of FOXO4 in these two cell lines. This result shows that miR-150 plays a role in negatively regulating FOXO4 expression by directly targeting its 3′UTR in NSCLC cells.

### Up-regulation of miR-150 and down-regulation of FOXO4 occur frequently in metastatic tumor cell lines and NSCLC tissues

**Figure 2:**
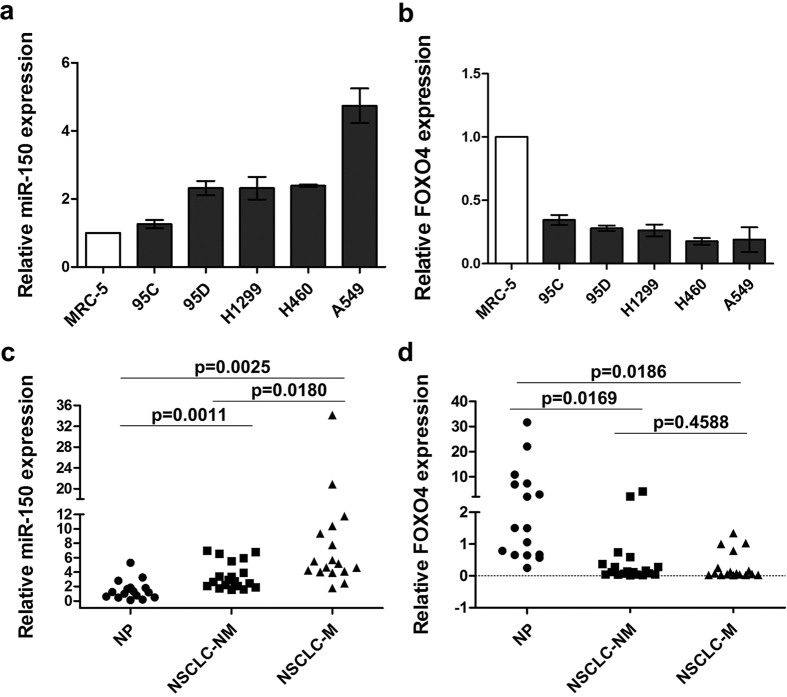
MiR-150 levels are inversely correlated with FOXO4 in human NSCLC tissues and cell lines. (**a**,**b**) Transcriptional levels of miR-150 (**a**) and FOXO4 (**b**) in human normal lung cell line (MRC-5) and five different NSCLC cell lines (95C, 95D, H1299, H460 and A549) were measured using qRT-PCR assay. Each bar represents the mean ± SD of three independent experiments. (**c**,**d**) Transcriptional levels of miR-150 (**c**) and FOXO4 (**d**) were measured in human NSCLC tissues including non-metastatic NSCLC tissues (NSCLC-NM, n = 19) and metastatic NSCLC tissues (NSCLC-M, n = 17), and non-neoplastic lung tissues (NP, n = 16) using qRT-PCR assay.

In order to investigate the correlation between miR-150-FOXO4 axis and metastasis in NSCLC, we first measured the expression of miR-150 and FOXO4 in five NSCLC cell lines (95C, 95D, H1299, H460 and A549) and normal human lung cell line (MRC-5). Among them, the paired low-metastatic 95C and high metastatic 95D cell lines were subcloned from a low differentiated human large cell lung carcinoma cell line PLA-801. H1299 and H460 cell lines were derived from lymph node and pleural effusion metastatic sites, respectively. The results showed that prominent higher expression of miR-150 transcript was observed in all five NSCLC cell lines while the expression of FOXO4 was dramatically down-regulated in all five NSCLC cell lines in comparison with the normal lung cell line (Fig. 2a and b). Specially, compared with 95C cells, expression of miR-150 was increased in high metastatic potential 95D cells which showed lower expression levels of FOXO4.

Further, to confirm the clinical significance of miR-150 and FOXO4 in NSCLC patients, we analyzed the transcriptional levels of miR-150 and FOXO4 in 16 cases of human non-neoplastic lung tissues and 36 cases of human NSCLC lung tissues including 19 cases of non-metastatic NSCLC tissues and 17 cases of metastatic NSCLC tissues by qRT-PCR assays. The results showed that compared to non-neoplastic lung tissues, miR-150 expression was frequently up-regulated in non-metastatic NSCLC tissues (*p* = 0.0011), especially in metastatic NSCLC tissues with higher expression levels (*p* = 0.0025) (Fig. 2c). In contrast, FOXO4 was frequently expressed at low levels in non-metastatic NSCLC tissues (*p* = 0.0169) and metastatic NSCLC tissues (*p* = 0.0186) (Fig. 2d). These data imply that the dysregulation of miR-150-FOXO4 axis is frequently observed in NSCLC progression, and FOXO4 and miR-150 potentially play opposite roles (pro-tumor and anti-tumor) in the tumor progression.

### Over-expression of miR-150 or knockdown of FOXO4 promoted NSCLC cell migration *in vitro*

**Figure 3:**
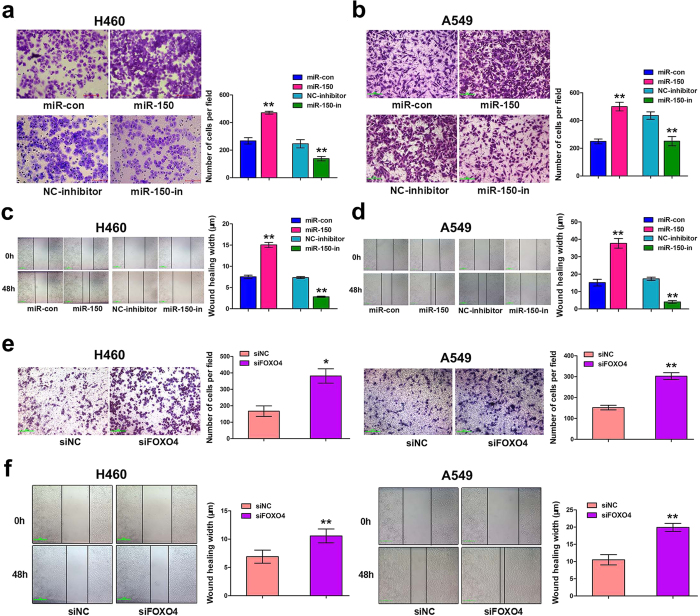
Over-expression of miR-150 and knockdown of FOXO4 promoted NSCLC cell migration *in vitro*. Representative images of the Transwell migration assay using H460 (**a**) and A549 (**b**) cells transiently transfected with pre-miR-150 plasmid/null vector and miR-150 inhibitor/NC-inhibitor. The cells penetrating the chamber membrane were shown. The results are expressed as the average number of cells in five random microscopic fields ± SD of three independent experiments. Representative images of the wound healing assay using H460 (**c**) and A549 (**d**) cells transfected with pre-miR-150 plasmid/null vector and miR-150 inhibitor/NC-inhibitor. The wound closure was quantified by measuring the distance between the invading front of cells using Image J. Experiments were performed in triplicate, and data are expressed as the mean ± SD. (**e**) Representative images of the Transwell migration assay using H460 and A549 cells transfected with siFOXO4 or siNC. (**f**) Representative images of the wound healing assay using H460 and A549 cells transfected with siFOXO4 or siNC. *p < 0.05 or **p < 0.01 versus the control. Scale bars: 20 μm.

To determine the role of miR-150-FOXO4 axis in NSCLC metastasis, transwell migration and wounding healing assay were performed in H460 and A549 cells. Pre-miR-150 plasmids were transfected into the cells and transfection efficiency was demonstrated by qRT-PCR (Supplementary Fig. S3). The results show that over-expression of miR-150 enhanced tumor cell migratory ability compared to empty vector transfected cells. As expected, knockdown of miR-150 suppressed cell migration ability (Fig. 3a,b,c and d). Consistent with the pro-metastatic effect caused by over-expression of miR-150, knockdown of FOXO4 by FOXO4 siRNA was also able to promote the migratory ability of H460 and A549 cells compared to corresponding siRNA NC-treated cells, as analyzed by the transwell migration and wound-healing assays (Fig. 3e and f ).

### Dysregulation of miR-150-FOXO4 signaling induced tumorigenesis and metastasis in mice

**Figure 4:**
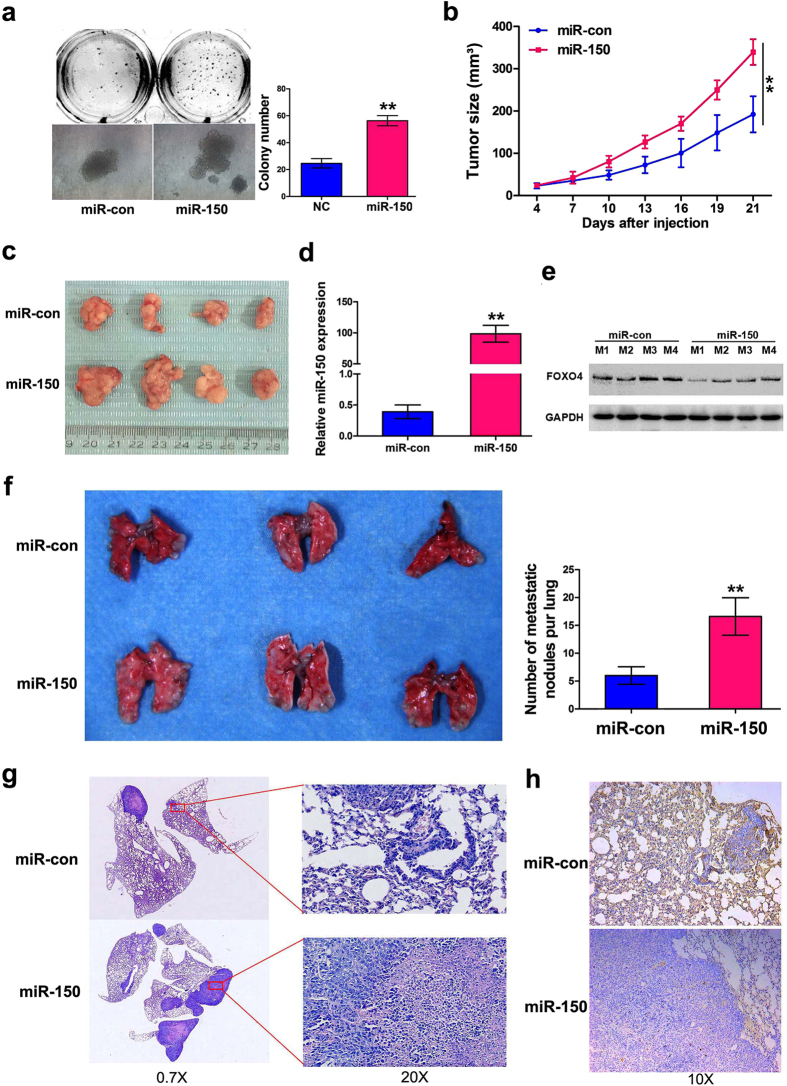
Dysregulation of miR-150-FOXO4 signaling induced tumorigenesis and metastasis in mice. (**a**) Colony growth of H460 stable cell lines in soft agar. Right panel, average number of colonies of H460 cells transfected with pre-miR-150 plasmid or null vector was evaluated 14 days after the seeding in soft agar. Results are shown as the mean ± SD of three independent experiments. (**b**) H460 cells stably transfected with pre-miR-150 plasmid and null vector were injected subcutaneously into the upper backs of the mice and tumor growth curves were record (*n* = 4 per group). (**c**) Representative images of xenograft tumors formed in nude mice are shown. (**d**) qRT-PCR analysis of miR-150 expression in xenograft tumor tissues. (**e**) Western blot analysis of FOXO4 protein expression in xenograft tumor tissues. A single band was detected at ~54 kDa, reflecting full-length expression. (**f**) H460 cells stably transfected with pre-miR-150 plasmid and null vector were injected into the tail veins of the mice (n = 5 per group). Representative images of the metastatic nodules of the lung (left). Numbers of visible metastatic nodules in individual mouse lungs (right). (**g**) Representative hematoxylin and eosin staining of tumor-bearing lung sections. Magnification ×0.7 (left); ×20 (right). (**h**) IHC analysis of FOXO4 expression in tumor-bearing lung sections, representative images were shown, magnification ×10. Data are shown as the mean ± SD, **p < 0.01.

Given that previous studies about proliferation-related role of miR-150 in lung cancer are mainly restricted to *in vitro* systems. However, the oncogenic effects of miR-150 are determined by its ability to induce tumorigenesis and/or metastasis *in vivo*. Therefore, an H460 cell line stably over-expressing miR-150 was established. We firstly evaluate the ability of the stable cell line to grow under anchorage independent condition *in vitro* using soft agar colony formation assays. As shown in the Fig. 4a, the H460-stably over-expressing miR-150 showed a significant increase in colony number compared to the control cell line, which indicating that the H460-stably over-expressing miR-150 is of a hallmark of malignant tumor cells *in vitro*. Further, the cells were subcutaneously or intravenously injected into nude mice. Representative results showed that subcutaneous tumors from the miR-150 over-expression group grew faster than those from the control group during tumor development (Fig. 4b). Consistently, the average tumor volume of the miR-150 over-expression group was significance larger that of the control group (Fig. 4c). Further, qRT-PCR and western blot analysis demonstrated that the stripped tumors from the miR-150 over-expression group had higher levels of miR-150 transcript and lower FOXO4 protein expression levels than those in the control group (Fig. 4d and e).

For intravenous inoculation, 30 days after injection, more lung metastatic nodules were observed in the lungs of the miR-150 over-expression group compared with the control group (Fig. 4f). H&E staining revealed that the metastatic incidence in the lungs of the miR-150 over-expression group were also markedly increased (Fig. 4g). Moreover, an obviously decrease in FOXO4 expression was also display in IHC slices of miR-150 over-expression group in comparison with the control group (Fig. 4h). These data establish a significant relevance between dysregulation of miR-150-FOXO4 signaling and significantly enhanced tumorigenesis and metastasis ability of NSCLC cells *in vivo*.

### Dysregulation of miR-150-FOXO4 signaling promoted tumor cell migration by stimulating EMT

**Figure 5:**
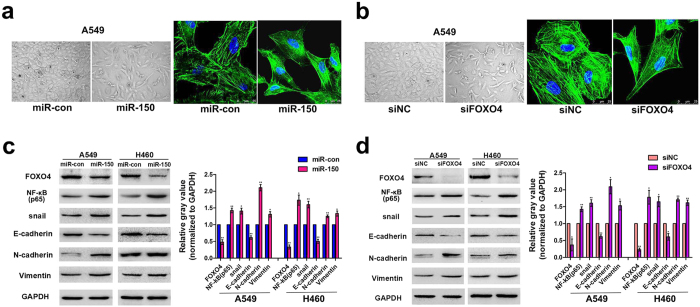
Dysregulation of miR-150-FOXO4 signaling promoted tumor cell migration by stimulating EMT. (**a**,**b**) The cell shapes of A549 cells were captured using a light microscope camera after the cells were transiently transfected with the miR-150 plasmid or vector control (*Left*), Scale bar, 50 μm. The cell morphology of A549 and H1299 cells was captured by confocal laser scanning microscope after the cells were transiently transfected with siFOXO4 or siNC, and stained with Alexa Fluor 488-labeled Phalloidin. Scale bars, 10 μm (*right*). (**c**,**d**) Western blot analysis of the expression of NF-κB, snail and E-cadherin, and the molecular markers of EMT, including N-cadherin and vimentin, in A549 and H460 cells transfected with pre-miR-150 plasmid or null vector (**c**) and siFOXO4 or siNC (**d**). GAPDH served as a loading control. Cropped blots are displayed. Right panel: densitometric analysis (protein quantification) and statistical analysis of protein bands using image J software. Statistical analyses of *n* = 3 independent experiments were assessed. *p < 0.05 or **p < 0.01 versus the loading control. Full-length blot can be seen in Supplementary Fig. S4. Supplementary Fig. S4a–c corresponds to Fig. 5c.

Activation of EMT has been shown to strongly promote tumor cell metastasis. To determine whether miR-150 is an EMT-regulatory miRNA in NSCLC, we first compared the cellular morphology change between miR-150 plasmid and empty vector- transfected A549 cells. Alexa Fluor 488-phalloidin was used to stain cytoskeletal F-actin in A549 cells. We found that the epithelial-like A549 cells with square or oval cell shapes were changed into a more spindle-like morphology with multiple cell protrusions (Fig. 5a). As a direct downstream target of miR-150, similar results were observed in A549 cells transfected with FOXO4 siRNA (Fig. 5b).

To evaluate whether the change in cell shape caused by dysregulation of miR-150-FOXO4 axis was associated with EMT-related molecules, we further examined the protein expression levels of the EMT markers E-cadherin, N-cadherin and vimentin. As expected, decreased expression levels of total E-cadherin and increased expression levels of N-cadherin and vimentin occurred in pre-miR-150 plasmid and FOXO4 siRNA-transfected A549 and H460 cells when compared to their corresponding counterpart (Fig. 5c and d). Changes of these EMT-related molecules may be the molecular representation of the phenotypic changes of tumor cells brought about as a consequence of dysregulation of miR-150-FOXO4 axis.

FOXO4 has been shown to suppress the transcriptional activity of nuclear factor-κB (NF-κB)^27^, a key component in the NF-κB/snail/YY1/RKIP circuitry. Deregulation of this circuitry is intimately involved in the initiation of EMT and tumor metastasis via up-regulation of snail, resulting in an inhibition of the epithelial marker E-cadherin^28^,^29^. Therefore, we detected the expression levels of NF-κB and snail in response to altered miR-150 or FOXO4 expression. Consistently, western blot results showed that over-expression of miR-150 or knockdown of FOXO4 was able to promote NF-κB and snail expression, further suppress E-cadherin expression (Fig. 5c and d). These results indicated that over-expression of miR-150 likely enhances EMT-like changes in NSCLC cells through FOXO4-mediated NF-κB/snail/YY1/RKIP circuitry regulation.

### Pro-metastatic effect of miR-150 was partially attributed to targeted silencing of FOXO4

**Figure 6:**
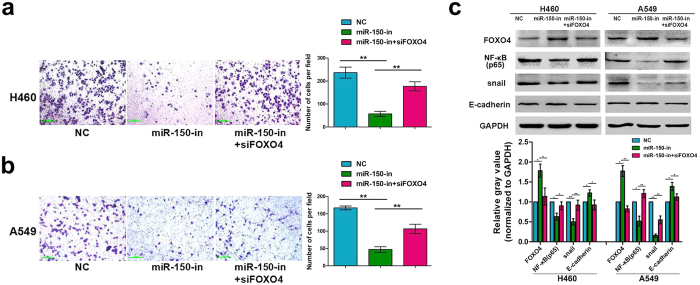
Pro-metastatic effect of miR-150 was partially attributed to targeted silencing of FOXO4. (**a**) Analysis of the migratory capacity of H460 cells transiently transfected with miR-150 inhibitor individually and co-transfected with the miR-150 inhibitor and siFOXO4 using the Transwell assay. (**b**) Analysis of the migratory capacity of A549 cells transiently transfected with miR-150 inhibitor individually and co-transfected with the miR-150 inhibitor and siFOXO4 using the Transwell assay. The mean number of migrated cells was counted from at least five randomly selected fields. Data are the mean ± SD of three independent experiments. **p < 0.01 versus the control. (**c**) Western blot analysis of the expression of FOXO4, NF-κB, snail and E-cadherin in H460 and A549 cells transiently transfected with miR-150 inhibitor individually and co-transfected with the miR-150 inhibitor and siFOXO4. Cropped blots are displayed, and densitometry analysis and statistical analysis of protein bands were shown on the right. Experiments were performed in triplicate, and data are expressed as the mean ± SD, *p < 0.05 or **p < 0.01 versus the control. Full-length blot can be seen in Supplementary Fig. S4. Supplementary Fig. S4c corresponds to (**c**).

To further confirm whether FOXO4 was directly involved in the pro-metastatic effect mediated by miR-150 in NSCLC, miR-150 inhibitor and FOXO4 siRNA were co-transfected into H460 and A549 cells to simultaneously silence miR-150 and FOXO4 expression. Impaired cell migration ability of H460 and A549 induced by the single-knockdown of miR-150 were partially restored by dual knockdown of FOXO4 and miR-150 (Fig. 6a and b). Moreover, up-regulation of E-cadherin, and down-regulation of NF-κB and Snail by single-knockdown of miR-150 were also reversed by dual knockdown of FOXO4 and miR-150 (Fig. 6c). These data show that FOXO4 is a key downstream metastatic effector of miR-150 and mediates, in part, the phenotypic and molecular effects of miR-150 in NSCLC.

## Discussion

Metastasis-related death accounts for approximately 90% of cancer mortality^30^. Accumulating evidences show that miRNAs participate in the tumor growth and/or metastastic process and a growing number of miRNAs have been found to be involved in lung cancer metastasis^5^,^31^. However, studies about the role of miR-150 in NSCLC are mainly confined to tumor growth and controversial in the limited published studies. Sun *et al*. showed that a significant decrease in miR-150 expression levels was found in tumors tissues compared with normal tissues or tumor-adjacent tissues^32^. However, another study reported that miR-150 was highly expressed in NSCLC and promoted the proliferation of tumor cells *in vitro*^7^. In this study, our findings support miR-150 functions as a pro-metastatic miRNA in NSCLC. Up-regulation of miR-150 was frequently observed in NSCLC metastatic tissues and metastatic tumor cell lines compared with adjacent non-tumor tissues or non-metastatic tissues and normal lung cell lines. Moreover, ectopic expression of miR-150 resulted in a significant increase in tumor cell metastasis *in vitro* and lung metastases in a nude mouse xenograft model.

The ability to regulate target gene expression allows miRNAs to regulate various biological processes including differentiation, proliferation, migration and apoptosis. *In vitro* assay demonstrated that miR-150 directly targets 3′-UTR of FOXO4, resulting in the suppression of FOXO4 expression. The functions of FOXO4 to induce cell cycle arrest, apoptosis and DNA-damage repair make it attractive candidates as tumor suppressors in human cancers^33^,^34^. For example, activation of FOXO4 reduces oncogene HER2-mediated tumor cell growth *in vitro* and *in vivo* through inhibiting AKT activity and maintaining p27^Kip1^ stability^35^. Previously, Li *et al*. reported that inactivation of FOXO4 inhibits the abilities of vascular smooth muscle cells to migrate *in vitro* through downregulation of MMP9^36^, suggesting that FOXO4 is also involved in cell migration process. In cancer, FOXO4 has been suggested to be a tumor metastasis suppressor. Su *et al*. showed that PI3K-AKT-mediated metastatic invasiveness in prostate cancer is associated with FOXO4 loss^26^. In gastric cancer, up-regulation of FOXO4 decreased tumor cell migration, accompanied by the downregulation of vimentin^25^. However, there has been very little research on the role of FOXO4 in lung cancer and whether FOXO4 has any metastasis effects in NSCLC remain unclear. Here, we report that FOXO4 is involved in the miR-150-induced tumor cell metastasis. Contrary to miR-150, down-regulation of FOXO4 were frequently observed in NSCLC clinical specimens and metastatic tumor cell lines. Knockdown of endogenous FOXO4 expression is able to promote tumor cell metastasis and hinder metastasis inhibition effects caused by inhibition of miR-150, suggesting that FOXO4 is a key downstream metastatic effector of miR-150.

EMT is one of the most commonly accepted cellular transitioning processes that drive tumor metastasis by which epithelial cells acquire mesenchymal, fibroblast-like properties including increased motility and decreased intercellular adhesion. EMT is under the control of miRNA networks^37^. Among FOXO family, Cheng *et al*. reported that FOXO3a-miR-622 axis inhibited HIF-1α to interfere mesenchymal characteristics of tumor cells in ERK-responsive lung cancer^38^. Liu *et al*. reported that FOXO3a-miR-34b/c axis restrains canonical β-catenin signaing-dependent EMT phenotypic changes^39^. Yang *et al*. reported that FOXO1 3′UTR can function as a ceRNA in repressing EMT and metastasis of breast cancer cells via regulating miR-9 activity^40^. For FOXO4, ANXA8 is transcriptionally down-regulated by EGF-mediated FOXO4 phosphorylation, which is correlated with the morphologic changes of EMT in the cholangiocarcinoma cells^41^. However, the relationship between miRNA-FOXO4 or FOXO4-miRNA axis and EMT has not yet been reported. Here, our results reveal that the dysregulation of the miR-150-FOXO4 axis may maintain tumor cells in a relatively undifferentiated state through EMT induction. We observed that A549 cells with over-expression of miR-150 or with knockdown of FOXO4 displayed morphological changes from a cobble stone-like to a spindle-like shape that is characteristic of mesenchymal cells when compared with their parental cells. Correspondingly, the mesenchymal markers N-cadherin, vimentin and snail were up-regulated while epithelial marker E-cadherin was down-regulated by miR-150 over-expression or FOXO4 knockdwon. Dysregulation of NF-κB/Snail/YY1/RKIP circuitry may be responsible for the EMT induction. A previous study showed that FOXO4 inhibited the transcriptional activity of NF-κB and that loss of FOXO4 induced NF-κB activity *in vivo*^27^, which is consistent with our observations that knockdown of FOXO4 or over-expression of miR-150 increased NF-κB expression, whereby resulting in an increase in snail expression and eventually promoting EMT progression.

Taken together, our study found that the dysregulation of miR-150-FOXO4 axis was frequently present in NSCLC, and miR-150 promotes cellular migration at least partially through its regulation of FOXO4 *in vitro* and *in vivo*. EMT-associated molecules (NF-κB, Snail, E-cadherin, N-cadherin and vimentin) potentially mediated pro-metastatic effects of the dysregulation of miR-150-FOXO4 axis. Therefore, miR-150-targeting interference combined with recovery of expression of FOXO4 may be a potential therapeutic strategy in metastatic NSCLC patients.

## Additional Information

**How to cite this article**: Li, H. *et al*. MiR-150 promotes cellular metastasis in non-small cell lung cancer by targeting FOXO4. *Sci. Rep.* **6**, 39001; doi: 10.1038/srep39001 (2016).

**Publisher's note:** Springer Nature remains neutral with regard to jurisdictional claims in published maps and institutional affiliations.

## Supplementary Material

Supplementary Information

## References

[b1] Siegel R., Ma J., Zou Z. & Jemal A. Cancer statistics, 2014. CA: a cancer journal for clinicians 64, 9–29, doi: 10.3322/caac.21208 (2014). 24399786

[b2] DeSantis C. E. . Cancer treatment and survivorship statistics, 2014. CA: a cancer journal for clinicians 64, 252–271, doi: 10.3322/caac.21235 (2014). 24890451

[b3] Shtivelman E. . Molecular pathways and therapeutic targets in lung cancer. Oncotarget 5, 1392–1433, doi: 10.18632/oncotarget.1891 (2014). 24722523 PMC4039220

[b4] Gasparini P. . microRNA expression profiling identifies a four microRNA signature as a novel diagnostic and prognostic biomarker in triple negative breast cancers. Oncotarget 5, 1174–1184, doi: 10.18632/oncotarget.1682 (2014). 24632568 PMC4012726

[b5] Hayes J., Peruzzi P. P. & Lawler S. MicroRNAs in cancer: biomarkers, functions and therapy. Trends in molecular medicine 20, 460–469, doi: 10.1016/j.molmed.2014.06.005 (2014). 25027972

[b6] He Y., Jiang X. & Chen J. The role of miR-150 in normal and malignant hematopoiesis. Oncogene 33, 3887–3893, doi: 10.1038/onc.2013.346 (2014). 23955084

[b7] Zhang N., Wei X. & Xu L. miR-150 promotes the proliferation of lung cancer cells by targeting P53. FEBS letters 587, 2346–2351, doi: 10.1016/j.febslet.2013.05.059 (2013). 23747308

[b8] Gu X. Y. . Down-regulation of miR-150 induces cell proliferation inhibition and apoptosis in non-small-cell lung cancer by targeting BAK1 *in vitro*. Tumour biology: the journal of the International Society for Oncodevelopmental Biology and Medicine 35, 5287–5293, doi: 10.1007/s13277-014-1688-4 (2014). 24532468

[b9] Huang S. . miR-150 promotes human breast cancer growth and malignant behavior by targeting the pro-apoptotic purinergic P2X7 receptor. PloS one 8, e80707, doi: 10.1371/journal.pone.0080707 (2013). 24312495 PMC3846619

[b10] Wu Q. . MiR-150 promotes gastric cancer proliferation by negatively regulating the pro-apoptotic gene EGR2. Biochemical and biophysical research communications 392, 340–345, doi: 10.1016/j.bbrc.2009.12.182 (2010). 20067763

[b11] Srivastava S. K. . MicroRNA-150 directly targets MUC4 and suppresses growth and malignant behavior of pancreatic cancer cells. Carcinogenesis 32, 1832–1839, doi: 10.1093/carcin/bgr223 (2011). 21983127 PMC3220613

[b12] Yokobori T. . MiR-150 is associated with poor prognosis in esophageal squamous cell carcinoma via targeting the EMT inducer ZEB1. Cancer science 104, 48–54, doi: 10.1111/cas.12030 (2013). 23013135 PMC7657108

[b13] Sun W. . MicroRNA-150 suppresses cell proliferation and metastasis in hepatocellular carcinoma by inhibiting the GAB1-ERK axis. Oncotarget 7, 11595–11608, doi: 10.18632/oncotarget.7292 (2016). 26871477 PMC4905496

[b14] Eijkelenboom A. & Burgering B. M. FOXOs: signalling integrators for homeostasis maintenance. Nature reviews. Molecular cell biology 14, 83–97, doi: 10.1038/nrm3507 (2013). 23325358

[b15] Kumar N. . Genome-wide endogenous DAF-16/FOXO recruitment dynamics during lowered insulin signalling in C. elegans. Oncotarget 6, 41418–41433, doi: 10.18632/oncotarget.6282 (2015). 26539642 PMC4747164

[b16] Furuyama T., Nakazawa T., Nakano I. & Mori N. Identification of the differential distribution patterns of mRNAs and consensus binding sequences for mouse DAF-16 homologues. The Biochemical journal 349, 629–634 (2000). 10880363 10.1042/0264-6021:3490629 PMC1221187

[b17] Fu Z. & Tindall D. J. FOXOs, cancer and regulation of apoptosis. Oncogene 27, 2312–2319, doi: 10.1038/onc.2008.24 (2008). 18391973 PMC2819403

[b18] Lam E. W., Shah K. & Brosens J. J. The diversity of sex steroid action: the role of micro-RNAs and FOXO transcription factors in cycling endometrium and cancer. The Journal of endocrinology 212, 13–25, doi: 10.1530/joe-10-0480 (2012). 21382987

[b19] Liu X. . MicroRNA-499-5p promotes cellular invasion and tumor metastasis in colorectal cancer by targeting FOXO4 and PDCD4. Carcinogenesis 32, 1798–1805, doi: 10.1093/carcin/bgr213 (2011). 21934092

[b20] Wang G. J., Liu G. H., Ye Y. W., Fu Y. & Zhang X. F. The role of microRNA-1274a in the tumorigenesis of gastric cancer: accelerating cancer cell proliferation and migration via directly targeting FOXO4. Biochemical and biophysical research communications 459, 629–635, doi: 10.1016/j.bbrc.2015.02.160 (2015). 25753202

[b21] Li J. . microRNA-150 promotes cervical cancer cell growth and survival by targeting FOXO4. BMC molecular biology 16, 24, doi: 10.1186/s12867-015-0052-6 (2015). 26715362 PMC4696189

[b22] Sun Z. . miR-150 inhibits terminal erythroid proliferation and differentiation. Oncotarget 6, 43033–43047, doi: 10.18632/oncotarget.5824 (2015). 26543232 PMC4767489

[b23] Wang Z. . Protein 4.1N acts as a potential tumor suppressor linking PP1 to JNK-c-Jun pathway regulation in NSCLC. Oncotarget 7, 509–523, doi: 10.18632/oncotarget.6312 (2016). 26575790 PMC4808014

[b24] Wang Z. . Knockout of 4.1B triggers malignant transformation in SV40T-immortalized mouse embryo fibroblast cells. Molecular carcinogenesis, doi: 10.1002/mc.22515 (2016). 27312663

[b25] Su L. . The transcription factor FOXO4 is down-regulated and inhibits tumor proliferation and metastasis in gastric cancer. BMC cancer 14, 378, doi: 10.1186/1471-2407-14-378 (2014). 24886657 PMC4063225

[b26] Su B. . A genome-wide RNAi screen identifies FOXO4 as a metastasis-suppressor through counteracting PI3K/AKT signal pathway in prostate cancer. PloS one 9, e101411, doi: 10.1371/journal.pone.0101411 (2014). 24983969 PMC4077825

[b27] Zhou W. . FoxO4 inhibits NF-kappaB and protects mice against colonic injury and inflammation. Gastroenterology 137, 1403–1414, doi: 10.1053/j.gastro.2009.06.049 (2009). 19560465 PMC2764529

[b28] Bonavida B. & Baritaki S. Dual role of NO donors in the reversal of tumor cell resistance and EMT: Downregulation of the NF-kappaB/Snail/YY1/RKIP circuitry. Nitric oxide: biology and chemistry/official journal of the Nitric Oxide Society 24, 1–7, doi: 10.1016/j.niox.2010.10.001 (2011). 20933602

[b29] Sanchez-Tillo E. . EMT-activating transcription factors in cancer: beyond EMT and tumor invasiveness. Cellular and molecular life sciences: CMLS 69, 3429–3456, doi: 10.1007/s00018-012-1122-2 (2012). 22945800 PMC11115078

[b30] Mehlen P. & Puisieux A. Metastasis: a question of life or death. Nature reviews. Cancer 6, 449–458, doi: 10.1038/nrc1886 (2006). 16723991

[b31] Leidinger P., Keller A. & Meese E. MicroRNAs - Important Molecules in Lung Cancer Research. Frontiers in genetics 2, 104, doi: 10.3389/fgene.2011.00104 (2011). 22303398 PMC3263430

[b32] Sun Y. . Expression of miR-150 and miR-3940-5p is reduced in non-small cell lung carcinoma and correlates with clinicopathological features. Oncology reports 29, 704–712, doi: 10.3892/or.2012.2152 (2013). 23228962

[b33] Greer E. L. & Brunet A. FOXO transcription factors in ageing and cancer. Acta physiologica (Oxford, England) 192, 19–28, doi: 10.1111/j.1748-1716.2007.01780.x (2008). 18171426

[b34] Sanchez A. M., Candau R. B. & Bernardi H. FoxO transcription factors: their roles in the maintenance of skeletal muscle homeostasis. Cellular and molecular life sciences: CMLS 71, 1657–1671, doi: 10.1007/s00018-013-1513-z (2014). 24232446 PMC11113648

[b35] Yang H., Zhao R., Yang H. Y. & Lee M. H. Constitutively active FOXO4 inhibits Akt activity, regulates p27 Kip1 stability, and suppresses HER2-mediated tumorigenicity. Oncogene 24, 1924–1935, doi: 10.1038/sj.onc.1208352 (2005). 15688030

[b36] Li H. . FoxO4 regulates tumor necrosis factor alpha-directed smooth muscle cell migration by activating matrix metalloproteinase 9 gene transcription. Molecular and cellular biology 27, 2676–2686, doi: 10.1128/mcb.01748-06 (2007). 17242183 PMC1899894

[b37] Bullock M. D., Sayan A. E., Packham G. K. & Mirnezami A. H. MicroRNAs: critical regulators of epithelial to mesenchymal (EMT) and mesenchymal to epithelial transition (MET) in cancer progression. Biology of the cell/under the auspices of the European Cell Biology Organization 104, 3–12, doi: 10.1111/boc.201100115 (2012). 22188537

[b38] Cheng C. W. . Foxo3a-mediated overexpression of microRNA-622 suppresses tumor metastasis by repressing hypoxia-inducible factor-1alpha in ERK-responsive lung cancer. Oncotarget 6, 44222–44238, doi: 10.18632/oncotarget.5826 (2015). 26528854 PMC4792553

[b39] Liu H. . FOXO3a modulates WNT/beta-catenin signaling and suppresses epithelial-to-mesenchymal transition in prostate cancer cells. Cellular signalling 27, 510–518, doi: 10.1016/j.cellsig.2015.01.001 (2015). 25578861

[b40] Yang J. . FOXO1 3′UTR functions as a ceRNA in repressing the metastases of breast cancer cells via regulating miRNA activity. FEBS letters 588, 3218–3224, doi: 10.1016/j.febslet.2014.07.003 (2014). 25017439

[b41] Lee M. J. . ANXA8 down-regulation by EGF-FOXO4 signaling is involved in cell scattering and tumor metastasis of cholangiocarcinoma. Gastroenterology 137, 1138–1150, 1150.e1131–1139, doi: 10.1053/j.gastro.2009.04.015 (2009). 19376120

