# Supplementary material for: MiR-150 promotes cellular metastasis in non-small cell lung cancer by targeting FOXO4
Source: Sci Rep. 2016 Dec 15;6:39001. doi: 10.1038/srep39001 (PMC5157020; doi:10.1038/srep39001)
Supplement: Supplementary Information [file srep39001-s1.pdf]

MiR-150 promotes cellular metastasis in non-small cell lung cancer by targeting FOXO4

Hui Li[1, #], Ruoyun Ouyang[2, #], Zi Wang[1, #], Weihua Zhou[1], Huiyong Chen[1], Yawen Jiang[1], Yibin Zhang[1], Hui Li[1], Mengting Liao[1], Weiwei Wang[6], Mao Ye[3], Zhigang Ding[4], Xueping Feng[5, *], Jing Liu[1, *], Bin Zhang[6, *]

1.The State Key Laboratory of Medical Genetics & School of Life Sciences, Central South University, Changsha 410078, China.

2. Department of Respiratory Medicine, Respiratory Disease Research Institute, Second XiangYa Hospital of Central South University, Changsha, 410011, China.

3.Molecular Science and Biomedicine Laboratory, State Key Laboratory for Chemo/Biosensing and Chemometrics, College of Biology, College of Chemistry and Chemical Engineering, Collaborative Innovation Center for Chemistry and Molecular Medicine, Hunan University, Changsha 410082, China.

4. Center for Experimental Medicine, the Third Xiangya Hospital, Central South University, Changsha 400083, China.

5. Institute of Medical Sciences, Xiangya Hospital,Central South University, Changsha 410008, China.

6.Department of Histology and Embryology, Xiangya School of Medicine, Central South University, Changsha 410013, China.

# These authors contributed equally to this work.

*** For correspondence:** Bin Zhang, e-mail address: coolzhangbin22@163.com; Jing Liu, e-mail address: jingliucsu@hotmail.com; Xueping Feng, e-mail address: fxp1029@aliyun.com.

Supplementary Table S 1. The list of primers and oligomers used in this study

FOXO4 siRNA sequence	5-CCUGGAGUGUGACAUGGAUAATT-3 (sense);
siRNA negative control (NC) sequence	5-UUCUCCGAACGUGUCACGUTT-3 (sense);
miR-150 inhibitor sequence	5-CACUGGUACAAGGGUUGGGAGA-3 (sense)
miR-150 inhibitor NC sequence	5-CAGUACUUUUGUGUAGUACAA-3 (sense)
miRNA mimic NC sequence	5-UUUGUACUACACAAAAGUACUG-3 (sense)
miR-150 mimics sequence	5-GUGACCAUGUUCCCAACCCUCU-3 (sense)
miR-421 mimics sequence	5-CGCGGGUUAAUUACAGACAACUA-3 (sense)
miR-499a-5p mimics sequence	5-UUUGUAGUGACGUUCAGAAUU-3 (sense)
miR-664a-3p mimics sequence	5-ACAUCCGACCCCUAUUUACUUAU-3 (sense)
FOXO4 primer sequence	5-CCUGGAGUGUGACAUGGAUAATT-3 (sense);
	5-UUAUCCAUGUCACACUCCAGGTT-3 (antisense);
Pre-miR-150 primer sequence	5-GAAGATCTTCTACTTTGCGCATCACACAGA-3 (sense);
	5-CCGCTCGAGCGGCCCTTGCTGGTTCTCTACTG-3 (antisense)
GAPDH primer sequence	5-CATGAGAAGTATGACAACAGCCT-3 (sense)
	5-AGTCCTTCCACGATACCAAAGT-3 (antisense);
FOXO4 WT 3'UTR primer sequence	5-AATTCTAGGCGATCGCTCGAGTATCTACTCTTTACCCTTGAGC-3 (sense)
	5-CAGCGGCCGCTCTAGGTTTAAACAAGCCTTTCTGTTATCTGC-3 (antisense);
FOXO4 mut 3'UTR primer sequence	5-CTGCCTGGAAACACAGGATTTTTTTGTAGAGA-3 (sense)
	5-CCTGTGTTTCCAGGCAGGGGTAAGGCCACTGG-3(antisense);

Supplementary Table S 2 . Information of antibodies

Western blotting antibodies	
	Anti-FOXO4 (sc-5221, Santa Cruz Biotechnology)
	Anti-NF-κB (6956, Cell Signaling Technology)
	Anti-snail (sc-10433, Santa Cruz Biotechnology)
	Anti-E-cadherin (3195, Cell Signaling Technology)
	Anti-N-cadherin (bs-4061s, Cell Signaling Technology)
	Anti-vimentin (sc-5565, Santa Cruz Biotechnology)
	Anti-GAPDH (sc-365062, Santa Cruz Biotechnology)
	Anti-ZEB1 (D80D3, Cell Signaling Technology)
	Anti-c-myb (ab117635, abcam)
	Anti-human 4.1R (kind gift of Xiuli An, New York Blood Center)
	Anti-PI3KCB (A0932, ABclonal)
Immunostaining antibodies	
	Alexa Fluor 488 Phalloidin (A12379, Invitrogene)

Supplementary figure

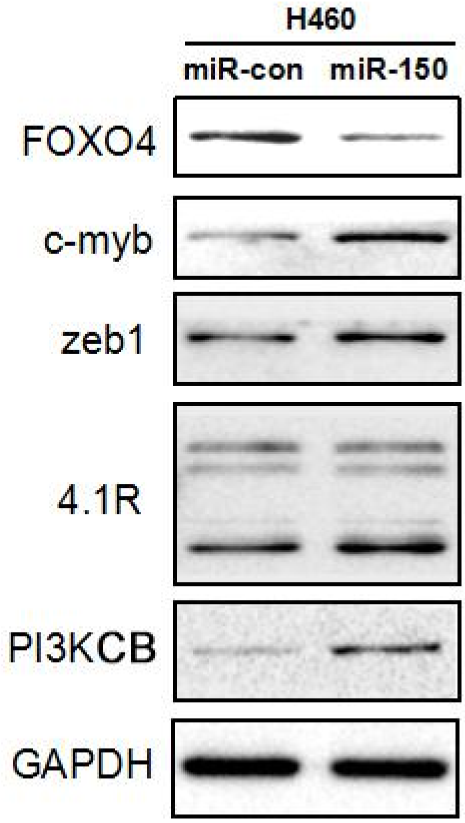

Fig.S1

Supplementary Fig. S1. Effects of miR-150 over-expression on the expression of several predicted targets of miR-150. Protein expression levels of candidate targets FOXO4, c-myb, Zeb1, 4.1R and PI3KCB in the H460 cells transfected with the pre-miR-150 plasmid or control vector. GAPDH served as a loading control.

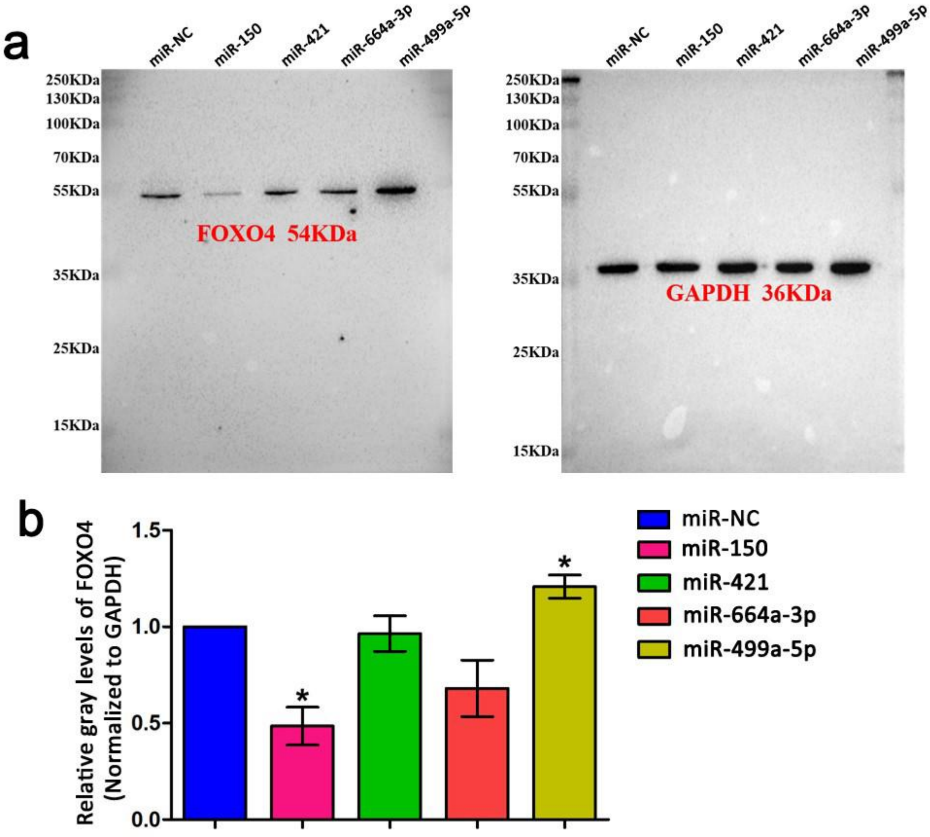

Fig.S2

Supplementary Fig. S2. Effects of several up-stream miRNAs of FOXO4 on the protein expression of FOXO4 in A549 cell lines. (a) Western blotting analysis of FOXO4 expression in A549 cells transfected with human-miR-150, -421, -664a-3p, -499a-5p mimics. miR-NC mimics were used as negative control. GAPDH served as the loading control. (b) Densitometric analysis of western blotting signals using image J software. Relative gray value in each protein level normalized to GAPDH is shown numerically. Statistical analyses of n=3 independent experiments were assessed, *p<0.05 versus control.

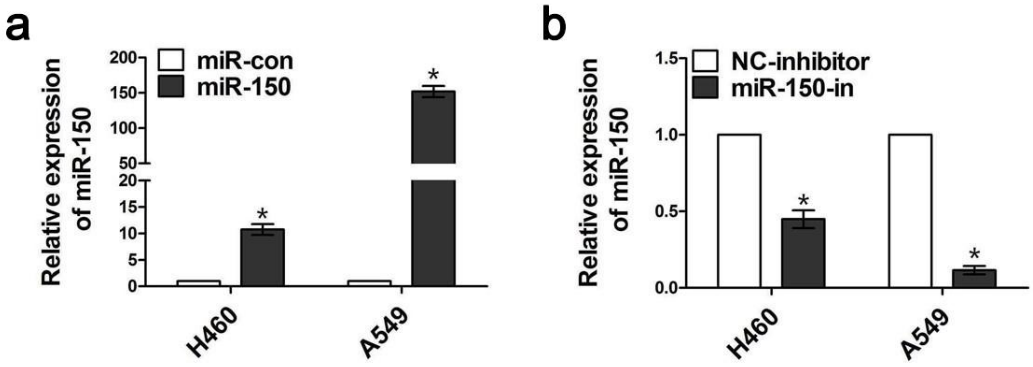

Fig.S3

Supplementary Fig. S3. qRT-PCR validation of changes in miR-150 expression levels in H460 and A549 cells after transfection. Relative fold changes in over-expression of miR-150 by transfection with pre-miR-150 plasmid (a) and relative fold changes in knockdown of miR-150 by miR-150 inhibitor (b) were evaluated by qRT-PCR. The expression of miR-150 in the cells transfected with null vectors or NC-inhibitors were regarded as the control. The results are shown as the mean ± SD of three independent experiments, **p* < 0.05.

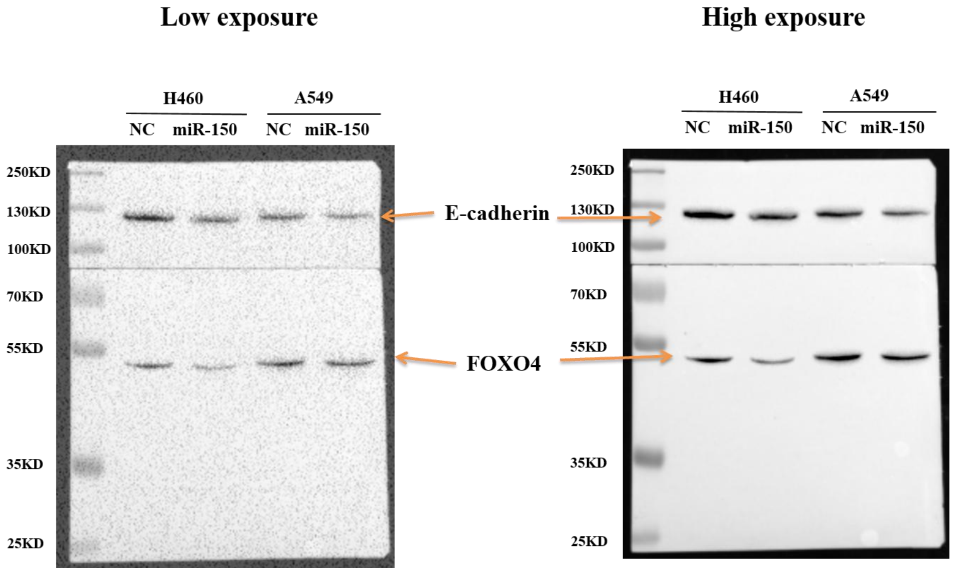

Fig. S4a

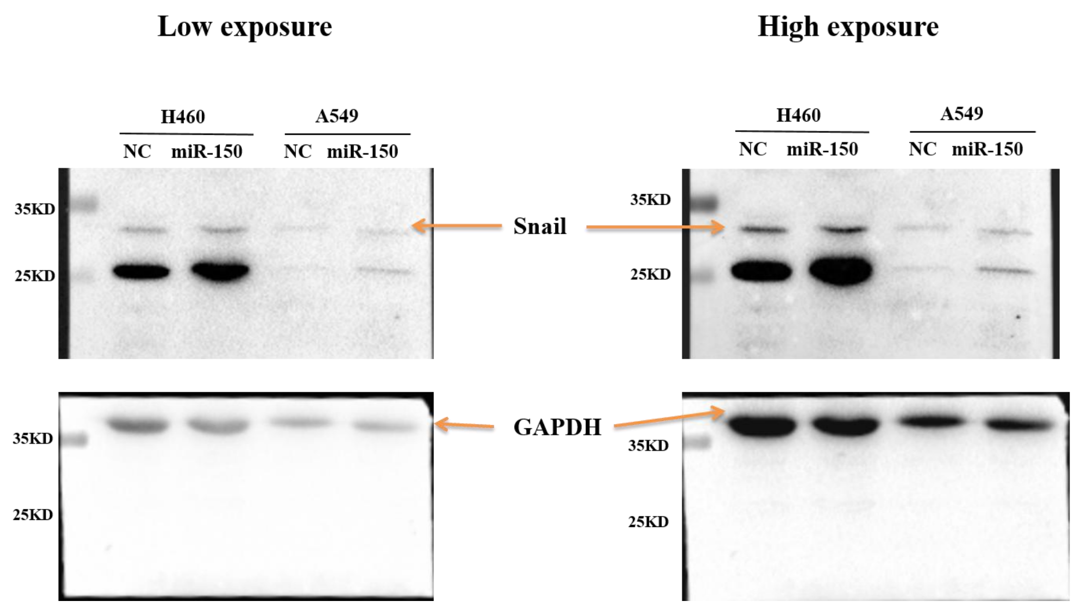

Fig. S4b

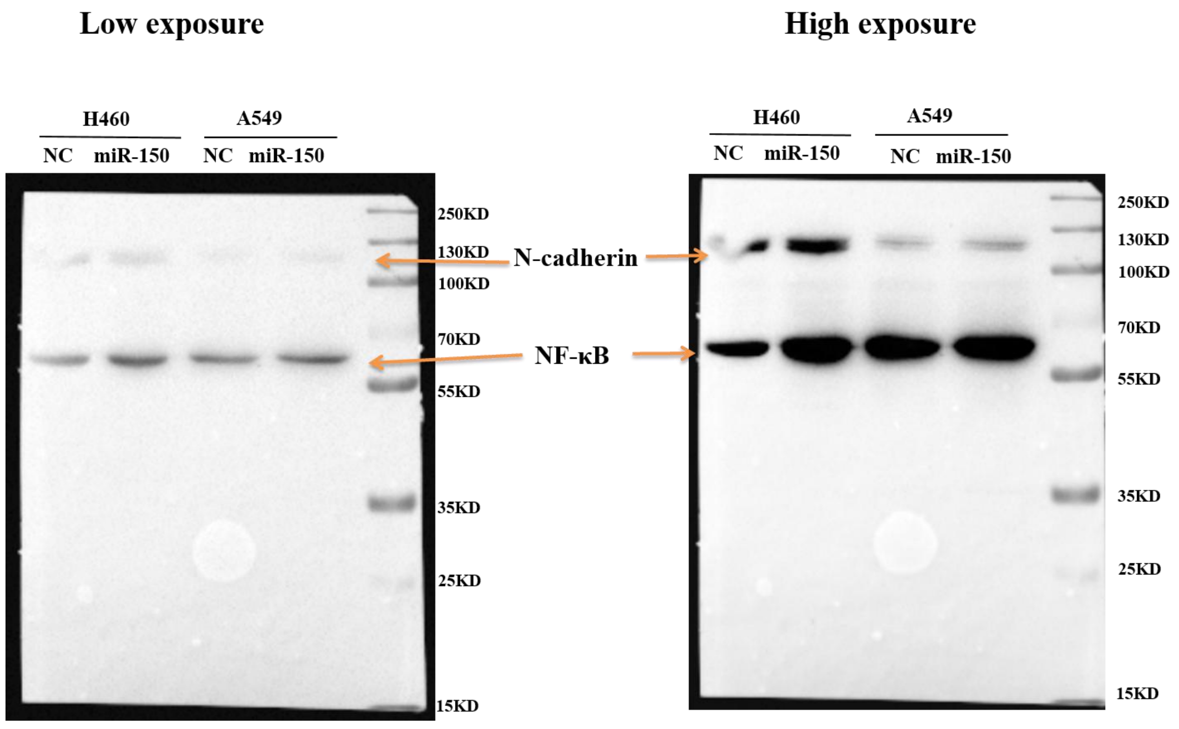

Fig. S4c

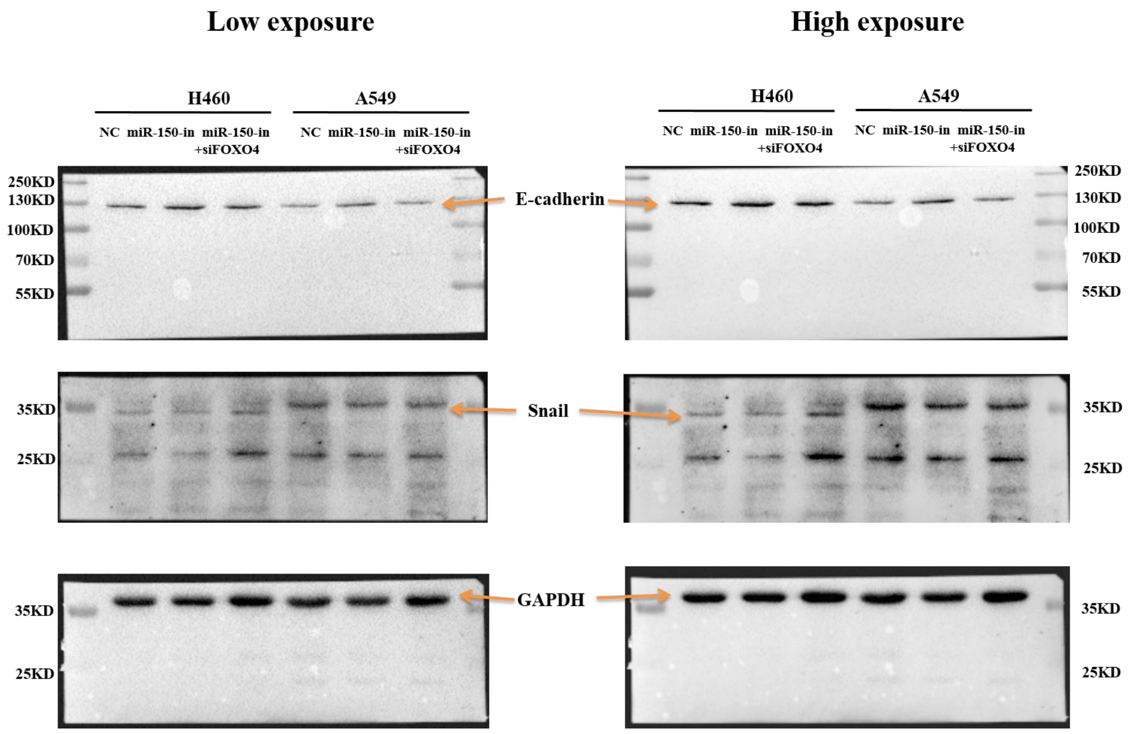

Fig. S4d

Supplementary Fig. S4. Full-length blots images. Full-length bot images for Fig.5c and Fig.6c.
